# Imaging Imageability: Behavioral Effects and Neural Correlates of Its Interaction with Affect and Context

**DOI:** 10.3389/fnhum.2016.00346

**Published:** 2016-07-15

**Authors:** Chris F. Westbury, Ivor Cribben, Jacqueline Cummine

**Affiliations:** ^1^Department of Psychology, University of AlbertaEdmonton, AB, Canada; ^2^Department of Finance and Statistical Analysis, University of AlbertaEdmonton, AB, Canada; ^3^Department of Communication Sciences and Disorders, University of AlbertaEdmonton, AB, Canada

**Keywords:** imageability, fMRI, lexical decision, semantics, affect, concreteness effect, lexical access, graphical models

## Abstract

The construct of *imageability* refers to the extent to which a word evokes a tangible sensation. Previous research (Westbury et al., 2013) suggests that the behavioral effects attributed to a word's imageability can be largely or wholly explained by two objective constructs, contextual density and estimated affect. Here, we extend these previous findings in two ways. First, we show that closely matched stimuli on the three measures of contextual density, estimated affect, and human-judged imageability show a three-way interaction in explaining variance in LD RTs, but that imagebility accounts for no additional variance after contextual density and estimated affect are entered first. Secondly, we demonstrate that the loci and functional connectivity (via graphical models) of the brain regions implicated in processing the three variables during that task are largely over-lapping and similar. These two lines of evidence support the conclusion that the effect usually attributed to human-judged imageability is largely or entirely due to the effects of other correlated measures that are directly computable.

## Introduction

The construct of *imageability*, the extent to which a word evokes a tangible sensation, has been the focus of much research in both typical populations (James, 1975; Strain et al., 1995; Hamilton and Rajaram, 2001; Westbury and Moroschan, 2009) and clinical populations (e.g., Goodglass et al., 1969; Warrington, 1975; Coltheart et al., 1980; Warrington and Shallice, 1984; Sirigu et al., 1991; Breedin et al., 1995; Cipolotti and Warrington, 1995; Marshall et al., 1996; Papagno et al., 2009). This work has demonstrated many behavioral effects, including effects on recall (e.g., Paivio, 1971, 1985, 1995; Hamilton and Rajaram, 2001), lexical access (e.g., James, 1975; Strain et al., 1995; Westbury and Moroschan, 2009), and age of acquisition (Bloom, 2000). High imageable words are recalled and accessed more quickly and learned earlier than low imageable words. In this paper, we follow up on previous work (Westbury et al., 2013), suggesting that the behavioral effects usually attributed to imageability may be attributed to correlated differences with two other well-defined constructs, contextual density, and estimated affect. We extend that work by demonstrating that the loci and degree of BOLD activation attributable to imageability, contextual density, and estimated affect constructs are highly correlated.

Previous imaging work has looked at both *imageability* and at *concreteness*, a closely-related construct. Westbury et al. (2013) noted that some non-concrete words (e.g., *eternal, glory, heaven*) have mid-range average imageability ratings, presumably because these words sometimes evoke tangible sensations. Conversely, some low frequency words with unambiguous concrete referents (e.g., *aster* and *astrolabe*) are rated on average as high on concreteness but low on imageability, presumably because raters know the referent is a real thing but are not exactly sure what it is. In this paper we treat imageability and concreteness as the same measure, for two reasons. One is that they are reliably correlated: *r* = 0.64, *p* < 0.00001, across the 1609 words for which Westbury et al. (2013) had ratings for both measures (compared to *r* = 0.81 for 1849 words that were rated independently by two groups for imageability). The second is that the words that dissociate on the measures seem to be a small and disparate set of special cases, rather than a coherent semantic category. We had both imageability and concreteness judgments for 64 of the 120 words in our stimulus set (described below), which are correlated at *r* = 0.87 (*p* < 0.00001). Although we use the term “imageability” throughout, we believe our results can be taken to apply equally to concreteness (but see Dellantonio et al., 2014 for a dissenting view).

## Theoretical accounts of imageability

Although there have been other attempts to account for the effects attributed to imageability, (e.g., Connell and Lynott, 2012), the most widely disseminated theoretical accounts of imageability are Paivio's (1971, 1985) *dual-coding theory*, and Schwanenflugel and colleagues' (Schwanenflugel and Shoben, 1983; Schwanenflugel and Stowe, 1989; Schwanenflugel, 1991) *context availability theory*. *Dual-coding theory* suggests that words are represented with two codes. One is a verbal code that can be used to represent words at both extremes of the imageability spectrum, from highly imageable to abstract. The other is a non-verbal code for representing imageable words that have associated sensory-motor information. According to dual coding theory, the more sensory-motor information associated with a word, the more highly imageable the word is. *Context availability theory*, on the other hand, proposes that imageability effects can be accounted for by a single system connecting words to their network of associated semantic knowledge. In this case, behavioral differences in accessing words at either end of the continuum reflect differences in the amount of information that links that semantic knowledge with each word, whether that knowledge comes from lexical semantics or real-world knowledge. Low imageable words are more dependent than high imageable words on contextual information (Schwanenflugel and Stowe, 1989).

Dual coding theory makes a fairly straightforward prediction about brain region involvement. Since the verbal code used for accessing abstract words is language-based, abstract words should make particular demands on the left hemisphere. The non-verbal code used for accessing imageable words, in virtue of being less reliant on language only, should make demands on both hemispheres. Imaging studies (e.g., Friederici et al., 2000; Jessen et al., 2000; Fiebach and Friederici, 2003; Noppeney and Price, 2004; Binder et al., 2005) have found some support for these predictions, although the results have been highly variable. Fiebach and Friederici (2003) presented a visual summary of studies that had used a variety of tasks to try to localize processing related to imageability (D'Esposito et al., 1997; Mellet et al., 1998; Kiehl et al., 1999; Friederici et al., 2000; Jessen et al., 2000; Wise et al., 2000). Perhaps the clearest general result was that the activity associated with imageable words was bilateral in most studies. More specifically, bilateral activity has been noted in the basal temporal lobe (Fiebach and Friederici, 2003) and parietal lobes, in addition to activity in the left inferior frontal lobe and left precuneous (Jessen et al., 2000) for concrete words. In contrast, activity for abstract words has been noted in the inferior frontal gyrus (Friederici et al., 2000; Fiebach and Friederici, 2003; Noppeney and Price, 2004), the left middle temporal lobe (Noppeney and Price, 2004), left postcentral sulcus, left posterior precuneus, and the right thalamus (Friederici et al., 2000). Overall, Noppeney and Price (2004) summarized the imageability literature by stating that “studies comparing abstract and concrete words have yielded inconsistent results and do not permit a clear interpretation because of additional task confounds” (p. 165).

One problem with all these studies is that lexical stimuli characteristics have been largely ignored, so stimulus words may differ on many dimensions. Binder et al. (2005) examined the role of concreteness using a lexical decision (LD) task with a stimulus set that matched words one-by-one on orthographic frequency, letter length, phoneme length, mean positional bigram frequency, and orthographic neighborhood size. They found that highly imageable words activated a bilateral network of regions that included the left and right angular and posterior cingulate gyri and precuneus, as well as the left dorsolateral prefrontal cortex. Non-imageable words in that study activated mainly a left hemisphere network, with peaks in the left precentral gyrus, superior temporal gyrus, and the middle and inferior frontal gyri. While Binder's et al. (2005) results are in line with hypotheses stemming from the dual coding theory more recent work has called into question the nature of human imageability judgments (Westbury et al., 2013), and subsequently, the validity of the dual coding theory to accommodate imageability effects.

Westbury et al. (2013) showed that studies whose imageability stimuli have been selected based on human imageability judgments (which is all studies considered thus far) are almost certain to be confounded along the dimensions of contextual density and estimated affect, making it impossible to know whether previously reported effects of imageability are true representations of the imageability construct.

### Contextual density and estimated affect

Westbury et al. (2013) proposed a different account of imageability effects building in part on the main insight of context availability theory–that low-imageability words are more context-bound than high-imageability words.

They used a co-occurrence model of lexical semantics to estimate the contextual density and relevant affect of words. In co-occurrence models, words are represented as vectors derived by computing how often each word co-occurs (within a small context window) with other words. Since it is possible to compute the similarity between two vectors using any one of a number of measures (in our case, the cosine between the vectors), the co-occurrence vectors for words therefore represent similarity of meaning from similarity of context of use. For example, such models can deduce that the word “cat” is more similar to the word “dog” than it is to the word “Marxism,” because the vectors for “dog” and “cat” will be more similar since these words tend to occur in similar contexts (i.e., with similar words). Since cosine distance is easy to compute for all vectors, it is easy to measure the similarity between a particular target word and all other words (or a subset of words) in the model. We used this to define our two measures of interest, contextual density and affective relevance, using Shaoul and Westbury's (2006a) HiDEx model of co-occurrence.

Contextual density (hereafter, DENSITY) was estimated using a linear combination of two measures—the mean similarity between the vector of the target word and the vectors of all other words within a pre-defined threshold (*Average Radius of Co-occurrence;* for details see Shaoul and Westbury, 2006a) and the number of words within that threshold (*N-count*)—weighted so they best predicted human imageability judgments.

Several lines of evidence suggest that affective associations are more important in processing abstract than concrete words (Altarriba et al., 1999; Kousta et al., 2011; Westbury et al., 2013). We estimated the role of affect using a linear combination of the co-occurrence distances of the vector for each each word from the vectors for eight affect terms, selected from a larger set of 78 terms using backwards regression on human imageability judgments. This measure has no simple interpretation outside of accounting for variance in human imageability judgments and we do not claim it is a simple valence or arousal measure^1^. To emphasize that it is a component of affect that is unique to predicting imageability judgments, we refer to it as *I-AFFECT*. More details about how these two measures are computed are given in Westbury et al. (2013).

DENSITY and I-AFFECT were entered into a linear regression model with additional lexical measures that are known to correlate with imageability judgments including word frequency and length (i.e., low imageability words are less frequent and longer, on average, than high imageability words: Reilly et al., 2012, 2016). Together these two measures produced estimates that correlated with the human imageability judgments on a validation set of 1849 words with *r* = 0.60 (*p* < 0.00001). When the predictions from this model were entered before human imageability judgments in an attempt to predict human LD response times on a pre-existing stimulus set, they accounted for 100% of the variance previously attributed to imageability judgments for that dataset, insofar as they blocked those judgments from entering into the model at all.

**Figure 1:**
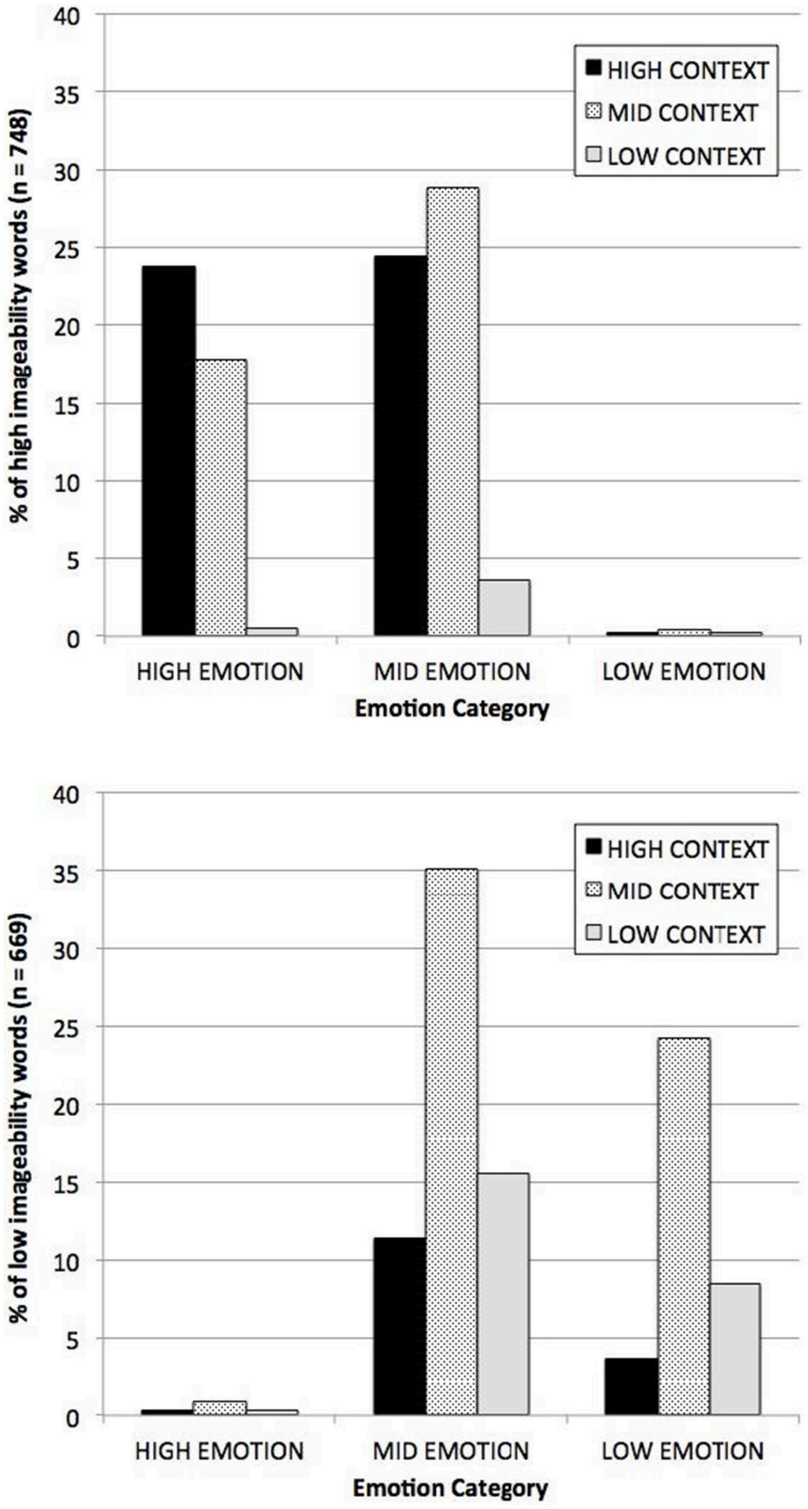
**Distribution among high imageable words (above) and low imageable words (below) of context and affect measures**. High and low groups were defined as outside ± 1 z, respectively. Reprinted from Westbury et al. (2013).

Of more direct relevance to the present study, Westbury et al. also showed that the distribution of I-AFFECT × DENSITY was radically asymmetrical across the range of words for which there are imageability judgments. In their sample of 3697 words, words rated by humans as high imageability were 80 times more likely than words rated as low imageability to be high (> 1SD [1z] from the mean) on I-AFFECT and DENSITY, whereas words rated as low imageability were 32 times more likely than word rated as high imageability words to be low (< −1z) on both measures (see Figure 1). As a result of this distributional asymmetry, researchers who select stimuli based on human imageability judgments are certain to be also systematically selecting them along the dimensions of DENSITY and I-AFFECT, resulting in a confound that makes it impossible to know whether effects that have been routinely attributed to imageability are actually due to imageability.

The implications of such a confound are substantial with respect to the theoretical accounts of imageability. In the current study, we explicitly examine the confound of imageability with DENSITY and I-AFFECT more closely in two experiments, a behavioral study and a functional magnetic resonance imaging [fMRI] study. We constructed a stimulus set for the LD task that was fully crossed (high/low) on all three dimensions, as outlined in more detail in the following section. This allowed us to compare behavioral and brain activation effects of each of the three predictors individually within a single set of subjects.

If imageability is indeed confounded with DENSITY and I-AFFECT, we expect to find evidence that there are no behavioral effects attributable to imageability after controlling for DENSITY and I-AFFECT, and to find evidence of over-lapping neural activation between imageability on the one hand, and DENSITY, and I-AFFECT on the other.

## Experiment 1: lexical decision

### Participants

Experiment 1 was carried out in accordance with the recommendations of the Human Research Ethics Board at the University of Alberta. All participants gave written informed consent in accordance with the Declaration of Helsinki. Participants included 132 undergraduate students (40 males). These participants were part of a larger multi-experiment study, not discussed here. As a result, every participant participated in four experimental tasks during their session, administered in random order. All participants self-reported to be right-handed and to have learned English natively before the age of 6 years. They had an average [SD] age of 19.6 [2.8] years, and an average [SD] of 1.8 [0.9] years of university education.

### Stimuli

We started with 3700 words of length 4 to 6 letters for which we have human imageability judgments from four published sources (Wilson, 1988; Bird et al., 2001; Cortese and Fugett, 2004; Stadthagen-Gonzalez and Davis, 2006, which is itself compiled from Paivio et al., 1968; Toglia and Battig, 1978; Gilhooly and Logie, 1980). Where we had multiple judgments on a word, we used the average judgment after normalizing to a 7-point scale. Because word frequency effects can mask imageability effects, the words were limited between 0.5 and 20 occurrences per million written words, using frequencies from Shaoul and Westbury (2006b).

We used the predictors described in detail in Westbury et al. (2013) to obtain estimated I-AFFECT and context measures. The I-AFFECT measure in this case is a linear combination of co-occurrence distances from eight affect terms (*admirable, arouse, envious, good, horny, pleasure*, and *proud*) that were identified as the best predictors of human imageability judgments by backward linear regression on 78 affect terms drawn from ten proposed models of “basic emotions,” as summarized in Appendix 1 (Supplementary Material). DENSITY is a linear combination of the two measure described above, ARC and N-COUNT. The models are fully specified in Westbury et al. (2013), and all the relevant measures (for 23,163 words with orthographic frequencies between 0.5 occurrences per million and 500 occurrences per million) are available for download from: http://www.psych.ualberta.ca/~westburylab/publications.html

We standardized the I-AFFECT and DENSITY estimates and the imageability judgments across all 3700 words, and removed all words that fell between –0.5 and +0.5 SD on any of the three measures. This guaranteed that high and low words on any of the three measures would differ by a minimum of 1 SD, a substantial difference. We undertook the matching by selecting the most extreme high/low words in each of the three categories that allowed us to define eight categories of 15 stimuli that fully crossed high/low in each category, a total of 8 categories x 15 words per category = 120 words, while closely matching on three lexical variables: logged orthographic frequency, word length, and orthographic neighborhood size. We matched words on these variables one by one, not on group averages, ensuring an extremely close match.

**Figure 2:**
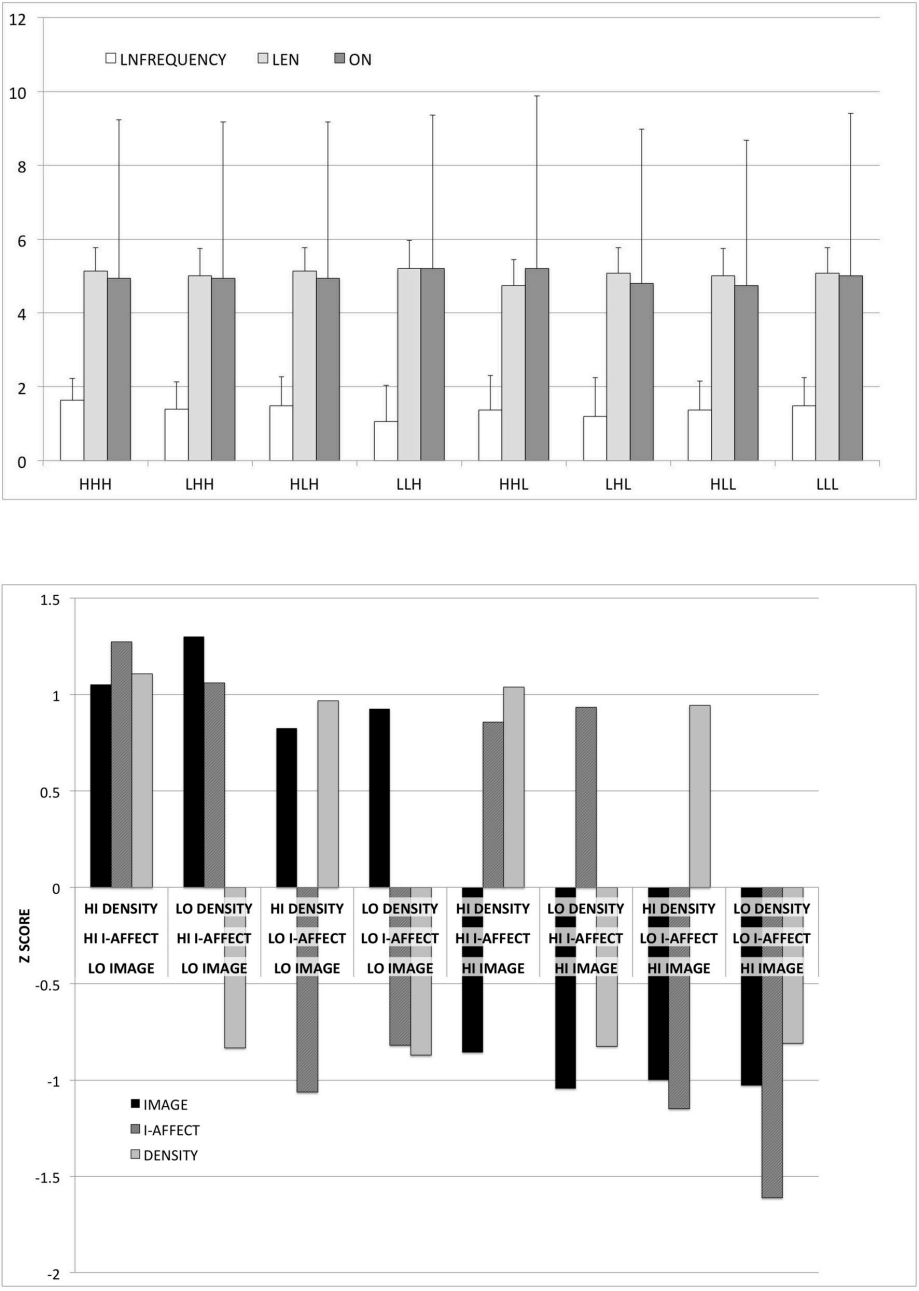
**Graphical illustration of stimuli properties. Above:** Logged orthographic frequency [LNFREQUENCY], length [LEN], and orthographic neighborhood size [ON], in each of the eight categories formed by crossing high/low imageability by affect by context. Bars show standard deviation. **Below:** Standardized imageability judgments [IMAGE], affect estimates [I-AFFECT], and co-occurrence density [DENSITY] estimates in each of the eight categories. Bars show standard deviation.

Because of its fully-crossed construction and word-by-word matching, this stimuli set can be split three ways, with 60 high and low words in each of the three categories, in such a way that after any such split, the two non-split categories will have an equal number of high and low words (30 each) in each the high and low categories of the split category, closely matched on the three lexical variables. The stimuli set is included in Appendix 2 (Supplementary Material). It is represented graphically in Figure 2, which illustrates that we successfully defined high/low categories that differed by roughly 2 SD on average within each of the three dimensions of interest, while tightly controlling for the lexical variables in which we have no interest here.

Each word was matched on length to a non-word (NW) generated by Markov chaining by three characters on an English dictionary using the freeware software LINGUA (Westbury et al., 2006, 2007). This method guarantees that the NWs conform to the same distribution of trigrams as real English words, and thereby produces readable, English-like NWs such as “yoot” and “whelf.”

### Procedure

Stimuli were presented to subjects using ACTUATE software (Westbury, 2007) running under Apple's OS 10.6 on G4 Mac Minis connected to 17″ LCD monitors, in a testing room constructed to reduce outside noise. Subjects were shown written instructions that were presented verbally at the same time by a research assistant. The instructions asked subjects to decide as quickly and accurately as they were able if each string was an English word, indicating their choice by pressing the “x” key (for “wrong”) or the “c” key (for “correct”). Strings were presented centered on the screen in a 312 pixel by 102 pixel white rectangle against a black background, in 60 point Times font. Each string was preceded by a “+” to orient the subjects to the coming stimulus, for a random amount of time between 500 and 1500 ms. The ISI was 1000 ms. Each subject began with two practice trials to familiarize them with the procedure. These trials were discarded before data analysis.

Data were trimmed by first removing all trials with reaction times [RTs] >3000 or <400 ms, and then removing all remaining stimuli that were more than 3 SDs [3 ^*^ 327 ms] slower than the average RT [774 ms.]. In total this removed 154 trials [0.46% of all trials] for being responded to too quickly and 1092 trials [3.29% of all trials] for being responded to too slowly.

### Results

The RT data were analyzed with R (R Core Team, 2013), using linear mixed-effect regression models fitted by Laplace approximation (Baayen, 2008), with Akaike Information Criterion [AIC] values used to adjudicate between models (Akaike, 1974). Models with lower AIC values are more likely to minimize information loss. We used a cut-off AIC difference of 5 as a criterion for choosing one model over another, corresponding to a difference of about 12 times in likelihood of minimizing information loss, although most differences were much larger.

We began the analysis by comparing models that included only single-predictor random effects for participant, stimulus order, age, gender, or years of education (see Table 1). The best single-predictor model contained a random effect for participant (AIC = 196457), which was slightly improved by also additively including a random effect for stimulus order (AIC = 196452). No improvements were found by including interactions between random effects. The lack of any effect of age and education may be attributable to the small variance in these measures.

**Table 1:** **Base Model analysis for LD RT**.

**Random effects**	**AIC**	**x participant**
Participant	196457	−>**196457**
Age	198179	196459
Gender	198206	196459
Order	198213	**196452**
Education	198216	196459
Fixed effects	AIC	
LnFrequency, Random Intercepts	**196289**	
Lnrequency, Random Intercepts and Subject Slopes	196287	
Length, Random Intercepts	196404	
Length, Random Intercepts and Subject Slopes	196404	
ON, Random Intercepts	196452	
ON, Random Intercepts and Subject Slopes	196449	
LnFrequency × ON, Random Intercepts	**196275**	
LnFrequency × ON, Random Intercepts and Subject Slopes	196274	
LnFrequency × ON × Length, Random Intercepts	**196138**	
LnFrequency × ON × Length, Random Intercepts and Subject Slopes	196213	

*Lower AIC values are better. Models that are an improvement over the previous best model (as determined by an AIC difference > = 5) are shown in bold*.

A base model was defined by adding lexical predictors that were not of direct theoretical interest in this study: word length, orthographic neighborhood size, and word frequency (the latter two computed using the frequency dictionary of Shaoul and Westbury, 2006b). As shown in Table 1, the best model (AIC = 196138) included an interaction term between all three predictors. We compared this model to a fixed effects only model, which contained the same three-way interaction but included no random effects. The fixed effects model had an AIC value of 197968 (AIC difference of 1830), confirming that the model with random effects is a much better fit to the data, millions of times more likely to minimize information loss.

As shown in Table 2, adding in the three predictors of interest, singly and in combination, has large effects on both the beta weights of the “irrelevant” lexical variables and on the intercept, making it problematic to do “head to head” comparisons of the weights on the predictors of interest. Nevertheless, there are two points relevant to understanding imageability in this table.

**Table 2:** **Model comparison for predicting LD RT**.

	**BASE**	**zI-AFFECT**	**zCONTEXT**	**zIMAGEABILITY**	**zI-AfFECT + zCONTEXT**	**zI-AFFECT × zCONTEXT +**	**zI-AfFECT + zIMAGEA.BILITY zCONTEXT**	zIAfFECT ^*^ zCONTEXT ^*^ zIMAGEABILITY [FinalModel1]	zI-AFFECT ^*^ zCONTEXT ^*^ zIMAGEA.BILITY [FinalModel2]
(Intercept)	726.78	696.08	750.21	728.80	720.79	721.56	744.62	807.38	832.30
LNFREQUENCY	61.27	91.38	[53.30]	[31.55]	81.34	80.59	[31.45]	[0.46]	−19.11
ON	29.08	33.28	32.52	19.21	36.18	36.21	23.80	18.97	8.80
LEN	[−2.32]	[2.7785]	[−6.31]	[−2.44]	[−1.4141]	[−1.47]	[−5.16]	[−15.38]	−20.35
LNFREQUENCY:ON	−17.62	−21.39	−20.84	[−8.83]	−24.12	−24.15	−13.08	[−7.93]	–
LNFREQUENCY:LEN	−14.52	−19.88	−13.05	[−9.21]	−18.04	−17.95	[−9.14]	[−3.89]	−2.68
ON:LEN	−6.05	−6.77	−6.94	−4.21	−7.54	−7.57	−5.29	−4.77	–
LNFREQUENCY:ON:LEN	3.10	3.78	3.85	[1.53]	4.43	4.46	2.47	[1.64]	–
zI-AfFECT	–	−7.68	–		−7.04	−6.90	[−1.80]	−3.80	−3.61
zCONTEXT	–	–	–12.10		−11.43	−11.44	−11.90	−15.16	−14.66
zIMAGEABILITY	–	–	–	−23.82	–		−23.28	−22.97	−23.19
zI-AFFECT x zCONTEXT	–	–	–	–	–	[−1.13]	–	−3.80	−3.05
zIMAGEA.BILITY × zCONTEXT		–	–	–	–	–	–	−22.97	7.09
zIMAGEA.BILITY × zI-AFFECT		–		–	–	–	–	−15.16	−3.64
zIMAGEABILITY × zI-AFFECT × zCONTEXT	–	–	–	–	–	–	–	9.02	8.91
AIC	196138	196102	196085	195877	196055	196053	195821	195737	195741
AIC - AIC[BASE]		−36	−53	−261	−83	−85	−317	−401	−397
CHI-SQUARE	N/A	32.05, *p* < 1.5e-08	46.64, *p* < 8.5e-12	145.25, *p* < 2.2e-16	79.1, *p* < 2.2e-16	88.7, *p* < 2.2e-16	216.5, *p* < 2.2e-16	331.8, *p* < 2.2e-16	294.8, *p* < 2.2e-16

*Predictors with weights in square brackets did not contribute reliably to the model. Lower AIC values indicate a better-fitting model. Models with random slopes had higher AIC values, so these models include only random intercepts. The final two columns compare models that included [FinalModel1] or did not include [FinalModel2] interactions of ON and length and ON with lnfrequency: i.e., FinalModel2 is a slightly simplified version of FinalModel1, with approximately the same IAC value (a difference < 5). The chi-square compares each model to the base model*.

One is that all three variables of interest are reliable predictors of RT when entered by themselves, as indicated by the large reduction in AIC over the base model in columns 3, 4, and 5 of Table 2.

The second point is that almost every time imageability is entered as a predictor, the effects of frequency and its interactions with length—which are otherwise always reliable—disappear from the model, as indicated by the fact that these predictors drop from the model in columns 5, 8, and 9 in Table 2. This suggests that imageability (but not I-AFFECT or DENSITY) is confounded with these lexical predictors.

Despite confounds between the predictors, the best model includes all the lexical predictors, plus imageability, DENSITY, and I-AFFECT in a three-way interaction. In keeping with the observation above, the first model with this three-way interaction model shows no reliable effect of frequency or its interaction with length or ON. An additional model that included no interactions of the lexical predictors with frequency was approximately equivalent by AIC (195741, vs. 195737 with all lexical predictors in interaction) but was judged better since it included no non-contributing predictors or interactions.

### Interim discussion

**Figure 3:**
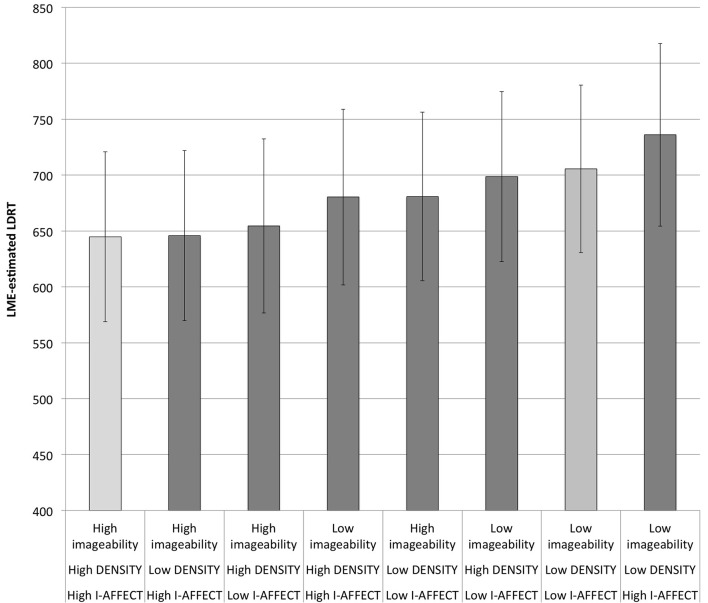
**Estimated LDRTs from the best LME model**. Light gray bars are the extreme cases, words that are all high or all low on imageability, DENSITY, and I-AFFECT. See also **Figure 4**.

The estimated RTs for that model are shown graphically in Figure 3, where they are sorted within the eight high/low imageability/DENSITY/I-AFFECT categories from shortest to longest RT. There are three main points to take away from this graph.

The first point is that the shortest RTs are seen in the four categories that are high on at least two out of three categories, and the longest RTs (by symmetry) are seen in the four categories that are low on at least two out of three categories: i.e., any two categories “pulling together” will trump a third category. In particular, there is no imageability effect (estimated difference of 0.4 ms) when low imageability words that are high on DENSITY and I-AFFECT are contrasted to high imageability words that are low on both DENSITY and I-AFFECT. One way of interpreting this result is to say that DENSITY and I-AFFECT together play an identical functional role in this task to imageability itself, replicating the main finding of Westbury et al. (2013).

The second point to take away is that the slowest category is not the category that is low on all three measures (which is second slowest, with an estimated average [SD] RT of 705.6 [74.9] ms) but rather the category that is low on imageability and context, but high on I-AFFECT (estimated average [SD] RT: 736.1 [81.7] ms). This may be because such words are “doubly unlikely,” since (as Westbury et al., 2013, pointed out) high I-AFFECT words are likely to also be high on both imageability and DENSITY.

The final, related point from Figure 3 has to do with the overall imageability effect. If we collapse across I-AFFECT and DENSITY, there is an estimated imageability effect of 48.7 ms. This apparently contradicts the original hypothesis that imageability effects in LD can be entirely explained by DENSITY and relevant affect. However, since there is (as noted above) no RT difference between low imageability words that are high on both DENSITY and I-AFFECT and high imageability words that are low on both DENSITY and I-AFFECT, the difference attributed to imageability may be due to collapsing over the remaining three cells nested within high and low imageability. These categories are also mismatched on the other two predictors, since the three remaining cells in each imageability category include two cells that are high on one non-imageability measure and low on the other. A better way to assess the “true” imageability effect is to account for variance attributable to DENSITY and I-AFFECT before looking at what remains to be explained by imageability. To do this, we re-ran the best model without including imageability (and dropping the interaction between DENSITY and I-AFFECT, which did not contribute reliably). In this model, the high imageability words showed estimated RTs of 675.4 ms, while the low imageability words showed estimated RTs of 684.6, for a negligible imageability effect of just 9 ms.

**Figure 4:**
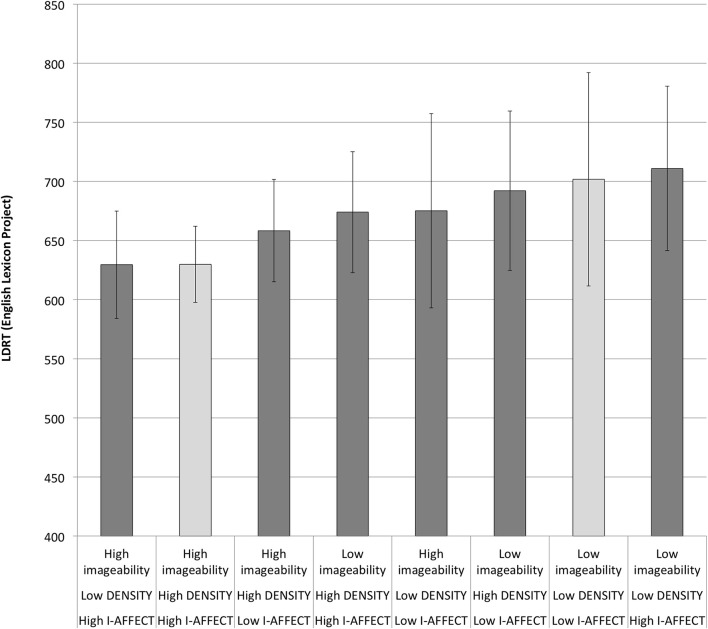
**LDRTs from the English Lexicon Project**. Light gray bars are the extreme cases, words that are all high or all low on imageability, DENSITY, and I-AFFECT. See also Figure 3.

We ran an analogous linear regression analysis (without using mixed effects models since we did not have subject-level data) using the average word RTs from the English Lexicon Project (Balota et al., 2007). The best model on this dataset was simpler, containing only imageability, plus an interaction between I-AFFECT and lnfrequency [*F*_(4, 114)_ = 11.7, *p* = 5.2e−08; *r*^2^ = 0.29]. The RTs are graphed in Figure 4. Although the models of the two datasets differ, the eight categories are ordered identically under both models, with the exception of a switch in the order of the first two categories (words high all three measures, vs. words high on just imageability, and affect), which is a minor difference since these categories have statistically nearly identical average estimated RTs under both models (645 vs. 646 ms in our data set; 630 vs. 629.5 in the ELP dataset).

We repeated the analysis above, whereby we accounted for variance attributable to DENSITY and I-AFFECT before looking at what remains to be explained by imageability. The best model for predicting LDRTs that did not contain imageability contained only lnfrequency and I-AFFECT. The fitted values from this model showed an imageability effect of just 4.8 ms (High imageability: 669.1 ms.; Low imageability 673.9 ms.), again a negligible effect consistent with the result reported above for our own data.

The mixed linear effects model is of course a much more reliable model than the linear regression on the averaged data, since it includes many more measures per category and controls for irrelevant random effects. We therefore conclude from these behavioral analyses that there is no effect on LD that is attributable to imageability itself after taking into account lexical differences in DENSITY and I-AFFECT.

## Experiment 2: fMRI of lexical decision

In the second experiment, we used fMRI to look at the changes in blood oxygen level dependent (BOLD) signals associated with each of the three predictors of interest.

### Participants

Fourteen university students (7 female; 7 male) participated in this study. None had participated in Experiment 1. Participants ranged in age from 18 to 22 years [Mean (SD) = 19.73 (1.33)] 12 were right-handed. Inclusion criteria consisted of normal or corrected-to-normal vision and English as a first language. Consent was obtained prior to data collection and according to the Declaration of Helsinki (World Medical Association, 2001). The experiment was conducted as part of a larger imaging protocol that included multiple reading-related experiments and was performed in compliance with the relevant laws and institutional guidelines and was approved by the host University Health Research Ethics Board. All participants were paid an honorarium.

### Image acquisition

Stimuli were presented using a data projector connected to the computer running E-Prime software (Psychology Software Tools, Inc., http://www.pstnet.com). Stimuli were delivered in black font on a white screen, in 36 Bold Courier New font, and appeared at the bottom center portion of a screen, which was visible to the participant through a mirror attached to the headcoil. Sixty fixation crosses were randomly interspersed with the 240 experimental trials resulting in an event-related design with a variable ITI, which allowed for a more accurate modeling of the subsequent impulse response function. The ISI was 2 s. Subjects were told they would see a series of words and non-words. They were asked to indicate, via a button press on an MRI compatible response pad, whether the stimulus was a word or a non-word, as quickly and accurately as possible.

Images were acquired on a 1.5T Siemens Sonata scanner and were positioned along the anterior-posterior commissure line.

Anatomical scans included a high-resolution axial T1 MPRAGE sequence with the following parameters: TR = 2000 ms, TE = 4.38 ms, number of slices = 112, base resolution 256 × 256, voxel size 1 × 1 × 1 mm, scan time 4:48 min. For the LD task, 310 volumes of 36 slice, axial spin, echo planar images (EPIs) were obtained with the following parameters: TR = 1970 ms, TE = 40 ms, base resolution 64 × 64 with a 128 × 128 reconstruction matrix that improved pixel resolution through zero-filling prior to Fourier transform reconstruction. EPI slice thickness was 4mm with no gap between slices.

### Data analysis

For each construct (imageability, DENSITY, I-AFFECT), we conducted the following analysis. The first five image volumes were used to achieve a steady state of image contrast and were discarded prior to analysis. The remaining 305 volumes were classified as words (120; 60 high and 60 low), non-words (120), or rest period (65) and were subject to standard pre-processing that was conducted using SPM8 (http://www.fil.ion.ucl.ac.uk/spm/). This included: realignment of images each other, slice timing correction, co-registration between functional and structural images, segmentation of maps into gray matter, white matter and cerebral spinal fluid, normalization into standard Montreal Neurological Imaging (MNI) space, and spatial smoothing using an 8 mm full width half maximum kernel.

Data were entered into a first level analysis using an event related design and a general linear model approach with two factors of interest (e.g., high construct words vs. low construct words) and six motion parameters of no interest. Estimation of the hemodynamic response function (HRF) was completed using restricted maximum likelihood (ReML) estimation, and activation for each participant was thresholded at *p* < 0.001 (no cluster-size correction). The following contrast maps were then created for each participant: (1) high construct words > non-words; (2) low construct words > non-words. Second level analysis included averaging of all participants to create a mean activation map for each contrast. Using a one-sample *t*-test, mean activation maps were significant at *t*_(13)_ = 3.85, *p* < 0.001. A cluster size threshold of 144 mm (18 voxels) was applied at the group level. In addition, a paired samples *t*-test was run to evaluate differences in activity between high and low construct conditions. Activation maps were significant at *t*_(13)_ = 3.85, *p* < 0.001, and an FDR cluster correction was applied to each map.

### Region of interest and graphical model analysis

**Figure 5:**
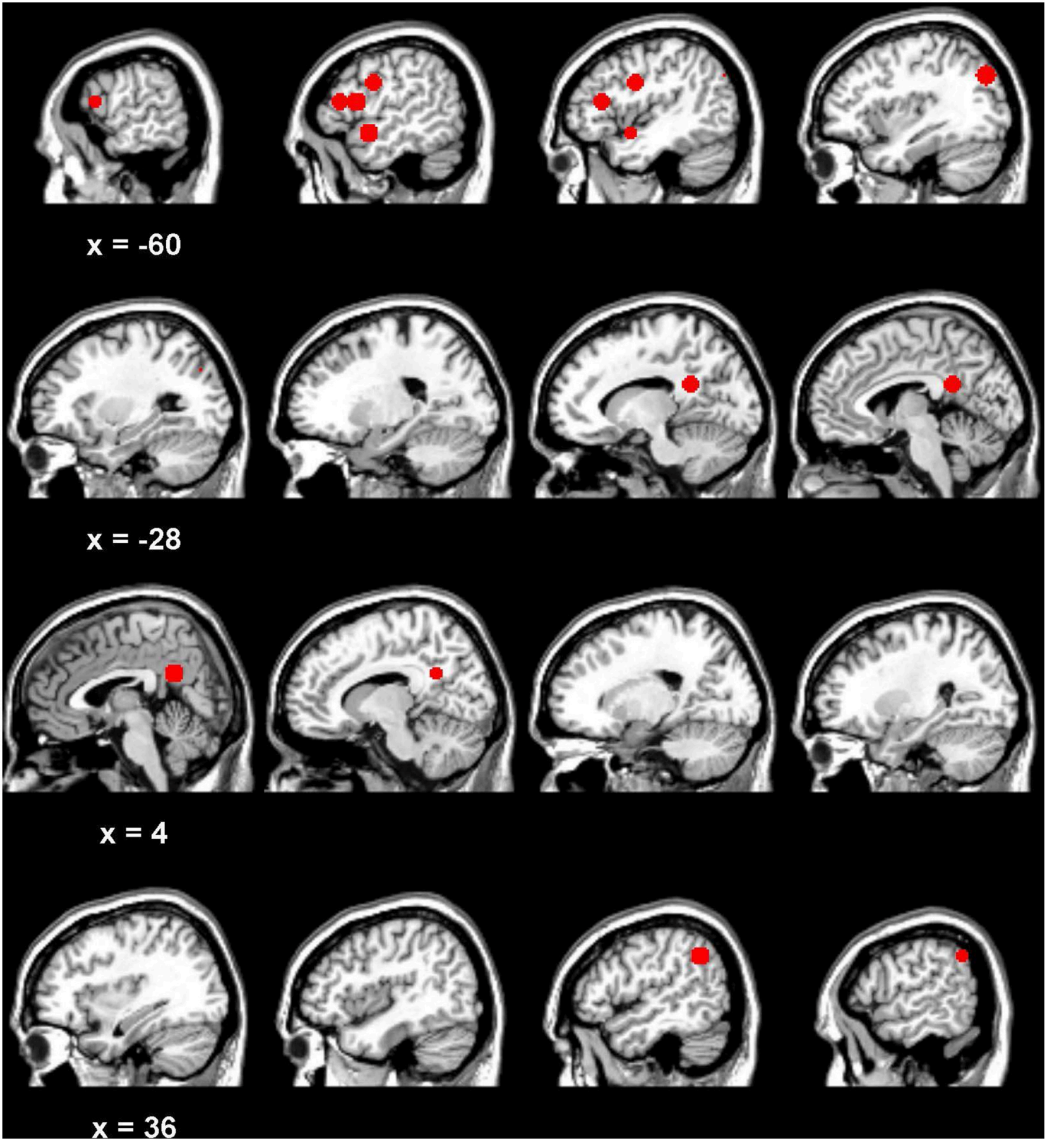
**Regions of interest for network analysis [see also **Figure 8**]**.

Eight regions of interest (10 mm spheres) were defined based on the main fronto-temporal regions of activity in the Binder et al. (2005) fMRI study of LD on high and low imageable words (see Table 3 and Figure 5): left and right angular gyrus, left and right posterior cingulate, left middle frontal gyrus, left inferior frontal gyrus, left precentral gyrus, and left superior temporal gyrus. These were drawn using MRIcron software (Rorden, 2005; http://www.mccauslandcenter.sc.edu/mricro/mricron). Regions were delineated on a standardized MNI template to which each participant's structural scan and functional scans were aligned. The global mean time series during the task blocks was then extracted using the Rex toolbox^2^ (Whitefield-Gabrieli, 2013; http://gablab.mit.edu/index.php/research/95-gablab-site/gablab/people/swg) for each participant in each region. The 120 word volumes for each participant for each construct were submitted for the graphical model estimation.

**Table 3:** **Identified regions of interest**.

**ROI**	***X***	***Y***	***Z***
Left angular gyrus	−36	−74	36
Right angular gyrus	54	−54	38
Left middle frontal gyrus	−49	29	15
Left posterior cingulate	−9	−46	24
Right posterior cingulate	6	−52	28
Left inferior frontal gyrus	−54	14	14
Left precentral gyrus	−48	1	30
Left superior temporal gyrus	−50	5	−12

Graphical modeling is a relatively recent technique in neuroimaging that can be used to study the functional connectivity or undirected association between pre-defined brain regions or voxel time courses (Albert and Barabási, 2002; Barabási and Bonabeau, 2003; Bullmore and Sporns, 2009; Smith et al., 2011). They are an ideal method for exploring brain networks in imageability as we have very little information on the interconnections between nodes in the network underlying imageability effects. Graphical models display the dependency structure between a set of ROIs using a graph *G* = (*V*,*E*), consisting of a set of vertices *V* and corresponding edges *E* that connect pairs of vertices. They may be defined as either undirected or directed with respect to how the edges connect one vertex to another. Directed graphs infer directionality between variables (or vertices) whereas undirected graphs do not. The current analyses used undirected graphs exclusively. In this case, each vertex represents a brain region and edges encode dependencies between the brain regions.

In order to estimate a graph, we first average the time courses across the voxels within each ROI. We then estimate the graph. The edges connecting ROIs represent the strength of connection (dependence) between the two ROIs. We estimated the undirected graph using the graphical lasso method (Friedman et al., 2008). Here an edge and missing edge between two vertices in the graph indicates a partial correlation and conditional independence between brain regions, respectively. The graphical lasso method assumes that the network structure is sparse which supports the idea of economic brain organization (Bullmore and Sporns, 2009). It is based on a penalized likelihood based on minimizing the Bayesian Information Criterion (BIC). After estimating the graph based on BIC minimization and identifying non-zero edges, the model was refit without the sparse inducing (or l_1_) constraint while keeping the zero elements in the matrix fixed to reduce bias and improve the model selection performance.

Because the graphical lasso is known to estimate a number of false positive edges in the estimated undirected graphs, we performed a bootstrap inferential procedure similar to the subsampling stability selection approach of Meinshausen and Bühlmann (2010). The goal was to control the family-wise type I multiple testing error. In this process, the data are bootstrapped many times and only edges that occurred in a large fraction of the resulting selection sets are retained. We used a bootstrap threshold, π_thr_, of 0.5 in the estimated undirected graphs in the figures. In other words, each edge in the undirected graphs was non-zero in 500 out of 1000 bootstrap samples of the data (for more details, see Cribben et al., 2012, 2013).

### Results

We briefly outline the results from these three closely-related perspectives of (1) BOLD signal analysis, (2) ROI signal comparison, and (3) network analysis. We defer discussion until we have presented results from all three analyses.

### Brain activity analysis

**Figure 6:**
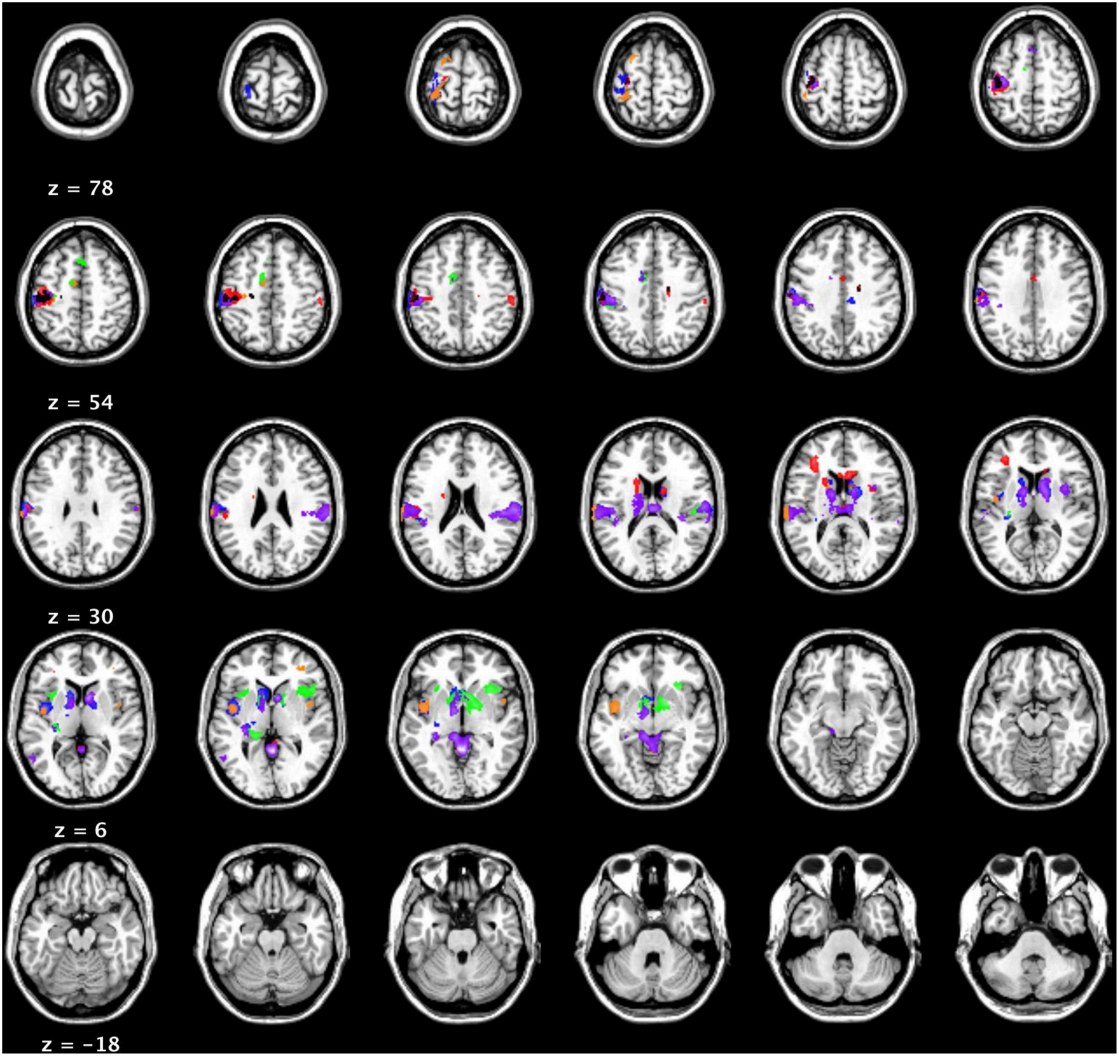
**fMRI results (***t*** = 3.85; cluster threshold of 144 mm = 18 voxels)**. Blue: High imageability > NW; Orange: Low imageability > NW; Red: High DENSITY > NW. Green: Low DENSITY > NW; Purple: High I-AFFECT >NW; Gold/Black: Low I-AFFECT > NW.

The BOLD data were analyzed using three subtractions, comparing words (>non-words) that were high or low on each of the three constructs of interest (imageability, DENSITY, I-AFFECT). Recall that the stimuli set was designed to be fully crossed high/low, so that splitting the data into high and low words by any one factor resulted in control of the other two factors. Each contrast is therefore highlighting BOLD signal changes due to the manipulation of a single factor of interest. The results are shown in Figure 6 and summarized in Table 4.

**Table 4:** **fMRI activation coordinates in Tailairach space**.

	***X***	***Y***	***Z***	**# voxels**	***t***	***p***	**LOCATION**
**MEAN ACTIVATION**							
High imageability (FDR cluster corrected at 49 voxels)	60	−18	22	188	8.26	< 0.001	Right inferior parietal lobule (BA 40)
	2	−50	−2	95	8.25		Right cerebellum
	10	6	14	327	8.19		Right caudate
	−40	−2	4	305	7.94		Left insula
	−54	−24	42	858	6.79		Left postcentral gyrus
	−58	−16	40		6.78		Left postcentral gyrus
	−62	−14	30		6.72		Left postcentral gyrus
	−16	0	4	439	6.08		Cingulate gyrus
	−12	12	6		6.01		Caudate
	−4	10	−4		5.95		Caudate
	−24	−34	74	296	5.54		Left postcentral gyrus
	−32	−36	70		5.35		Left postcentral gyrus
	−26	−42	72		5.02		Left postcentral gyrus
	−28	−28	10	99	5.39		Thalamus
Low Imageability (FDR cluster corrected at 41 voxels)	−66	−30	18	182	6.66	< 0.001	Left superior temporal gyrus
	−66	−18	20		5.35		Left postcentral gyrus
	−56	−18	12		4.76		Left superior temporal gyrus
	60	−14	20	95	6.15		Right postcentral gyrus
	−22	6	66	43	6.09		Left superior frontal gyrus
	−32	−22	60	521	5.77		Left precentral gyrus
	−40	−24	56		5.30		Left postcentral gyrus
	−28	−34	68		5.26		Left postcentral gyrus
	2	−50	−4	105	5.66		Culmen
	4	−40	−4		4.63		Culmen
	−42	−2	−4	240	5.62		Left insula
	−42	−4	6		4.82		Left insula
	−10	−2	−4	51	5.43		Lentiform Nucleus
	−16	−2	16	81	5.18		Cingulate gyrus
	−8	−2	50	59	4.93		Cingulate gyrus
	44	2	4	41	4.81		Right insula
High DENSITY (FDR cluster corrected at 86 voxels)	2	−48	−2	173	8.49	< 0.001	Culmen
	−20	14	16	86	7.35		Caudate
	−20	−4	24		5.04		Caudate
	−16	2	18		4.56		Caudate
	−62	−18	30	1154	6.96		Left postcentral gyrus
	−52	−26	50		6.64		Left inferior parietal lobule (BA40)
	−42	−24	54		5.79		Left postcentral gyrus
	−32	36	14	130	6.26		Middle frontal gyrus
	−34	26	16		6.25		Left insula
	6	20	12	86	5.95		Caudate
	14	24	14		5.14		Anterior cingulate
Low DENSITY (FDR cluster corrected at 87 voxels)	16	2	−4	677	7.73	< 0.001	Leniform Nuclues
	10	10	−2		6.33		Caudate
	−6	−4	−6		6.03		Hypothalamus
	60	−18	20	170	7.23		Right postcentral gyrus
	48	−22	18		5.25		Right insula
	32	20	−4	236	6.52		Claustrum
	48	18	−2		5.79		Right insula
	−20	−32	2	110	5.85		Thalamus
	−28	−24	6		5.11		Thalamus
	−10	2	44	131	5.79		Cingulate gyrus
	−12	8	40		4.58		Cingulate gyrus
	−10	−4	52		4.51		Medial Frontal gyrus
	−30	14	4	87	5.54		Claustrum
	−28	22	−4		4.73		Claustrum
	−34	−18	62	105	5.25		Precentral gyrus
High I-AFFECT (FDR cluster corrected at 99 voxels)	2	−50	0	474	10.36	< 0.001	Culmen
	−6	−38	−4		7.56		Culmen
	10	10	6	1036	7.36		Caudate
	−16	−26	16		6.76		Thalamus
	14	2	10		6.43		Caudate
	48	−24	24	515	6.99		Right insula
	64	−24	24		6.70		Right inferior parietal lobule (BA40)
	60	−18	20		6.53		Right postcentral gyrus
	−48	−24	44	1042	6.25		Left postcentral gyrus
	−34	−2	60		6.14		Left precentral gyrus
	−64	−20	30		5.68		Left inferior parietal lobule (BA40)
	−30	−28	2	99	6.10		Thalamus
Low I-AFFECT (FDR cluster corrected at 309 voxels)	−56	−22	46	309	5.39	< 0.001	Left postcentral gyrus
	−36	−18	62		4.88		Left precentral gyrus
	−50	−26	54		4.30		Left postcentral gyrus
**SUBTRACTION**							
High I-AFFECT - NW > Low I-AFFECT - NW	−17	16	2		4.02	0.001	L. Caudate Head
High I-AFFECT - NW > Low I-AFFECT - NW	−11	−27	27		4.15	0.001	L. Caudate Tail
High I-AFFECT - NW > Low I-AFFECT - NW	−20	16	4		4.2	0.001	L. Putamen
High I-AFFECT - NW > Low I-AFFECT - NW	41	−69	17		4.45	0.0001	R. Brodmann area 39
High I-AFFECT - NW > Low I-AFFECT - NW	40	−73	15		3.95	0.001	R. Brodmann area 39
High I-AFFECT - NW > Low I-AFFECT - NW	17	14	6		4.03	0.001	R. Caudate Body
Low DENSITY - NW > High DENSITY - NW	−4	14	43		5.13	0.0001	L. Brodmann area 32
Low DENSITY - NW > High DENSITY - NW	−40	−48	−13		5.46	0.0001	L. Brodmann area 37
Low DENSITY - NW > High DENSITY - NW	−24	3	47		5.05	0.0001	L. Brodmann area 6
Low DENSITY - NW > High DENSITY - NW	−25	18	−3		5.56	0.0001	L. Claustrum
Low DENSITY - NW > High DENSITY - NW	−6	−5	−6		4.75	0.0001	L. Hypothalamus
Low DENSITY - NW > High DENSITY - NW	40	18	−1		4.23	0.0001	R. Brodmann area 13
Low DENSITY - NW > High DENSITY - NW	33	−79	17		4.25	0.001	R. Brodmann area 19
Low DENSITY - NW > High DENSITY - NW	19	−54	15		4.03	0.001	R. Brodmann area 30
Low DENSITY - NW > High DENSITY - NW	29	−56	29		4.08	0.001	R. Brodmann area 39
Low DENSITY - NW > High DENSITY - NW	8	12	−3		4.83	0.0001	R. Caudate Head
Low DENSITY - NW > High DENSITY - NW	12	−1	−6		4.64	0.0001	R. Medial Globus Pallidus
Low DENSITY - NW > High DENSITY - NW	17	−5	−2		3.86	0.001	R. Medial Globus Pallidus
Low DENSITY - NW > High DENSITY - NW	26	16	−3		5.91	0.0001	R. Putamen
Low DENSITY - NW > High DENSITY - NW	33	−15	2		4.12	0.001	R. Putamen
Low Imageability - NW > High Imageability -NW	−45	−44	−8		4.33	0.0001	L. Brodmann area 37
Low Imageability - NW > High Imageability -NW	−13	−36	61		3.86	0.001	L. Brodmann area 4
Low Imageability - NW > High Imageability -NW	−20	39	23		4.02	0.001	L. Brodmann area 9
Low Imageability - NW > High Imageability -NW	−15	41	25		3.98	0.001	L. Brodmann area 9
Low Imageability - NW > High Imageability -NW	−25	−32	−21		4.09	0.001	L. Culmen
Low Imageability - NW > High Imageability -NW	−6	−29	11		3.88	0.001	L. Pulvinar
Low Imageability - NW > High Imageability -NW	−4	−21	−3		5.13	0.0001	L. Red Nucleus
Low Imageability - NW > High Imageability -NW	8	30	40		3.97	0.001	R. Brodmann area 8
Low Imageability - NW > High Imageability -NW	33	−44	10		4.37	0.0001	R. Hippocampus
Low Imageability - NW > High Imageability -NW	1	−1	1		4.18	0.001	R. Thalamus
High Imageability - NW > Low Imageability -NW	−50	−52	6		4.26	0.0001	L. Brodmann area 39

The mean activation maps associated with imageability, DENSITY and I-AFFECT showed similar patterns of activity with common activation in the following regions: anterior/medial cingulate, left insula, left pre, and post-central gyri, and bilateral subcortical regions of the basal ganglia structures. We found just a single left hemisphere region of activation associated with high imageability [(HI – NW) – (LI – NW)], at –50, –52, 6 in BA 39 in the angular gyrus. The inverse contrast [(LI – NW) – (HI – NW)] showed two reliable clusters at BA 9 in the left medial frontal gyrus (–20, 39, 23; –15, 41, 25), a cluster in BA 4 in the left postcentral gyrus (–13, –36, 61) and a cluster in BA 8 in the right medial frontal gyrus (8, 30, 40). Low imageability words also showed activation in the left thalamus (–6, –29, 11), the mid thalamus (1, –1, 1), the left red nucleus (–4, –21, –2), and the left cerebellum (–25, –32, –21).

### ROI analysis

A series of paired samples *t*-tests were used to test for differences in brain activity across each of the conditions of interest, in each of the eight ROIs mentioned above.

**Figure 7:**
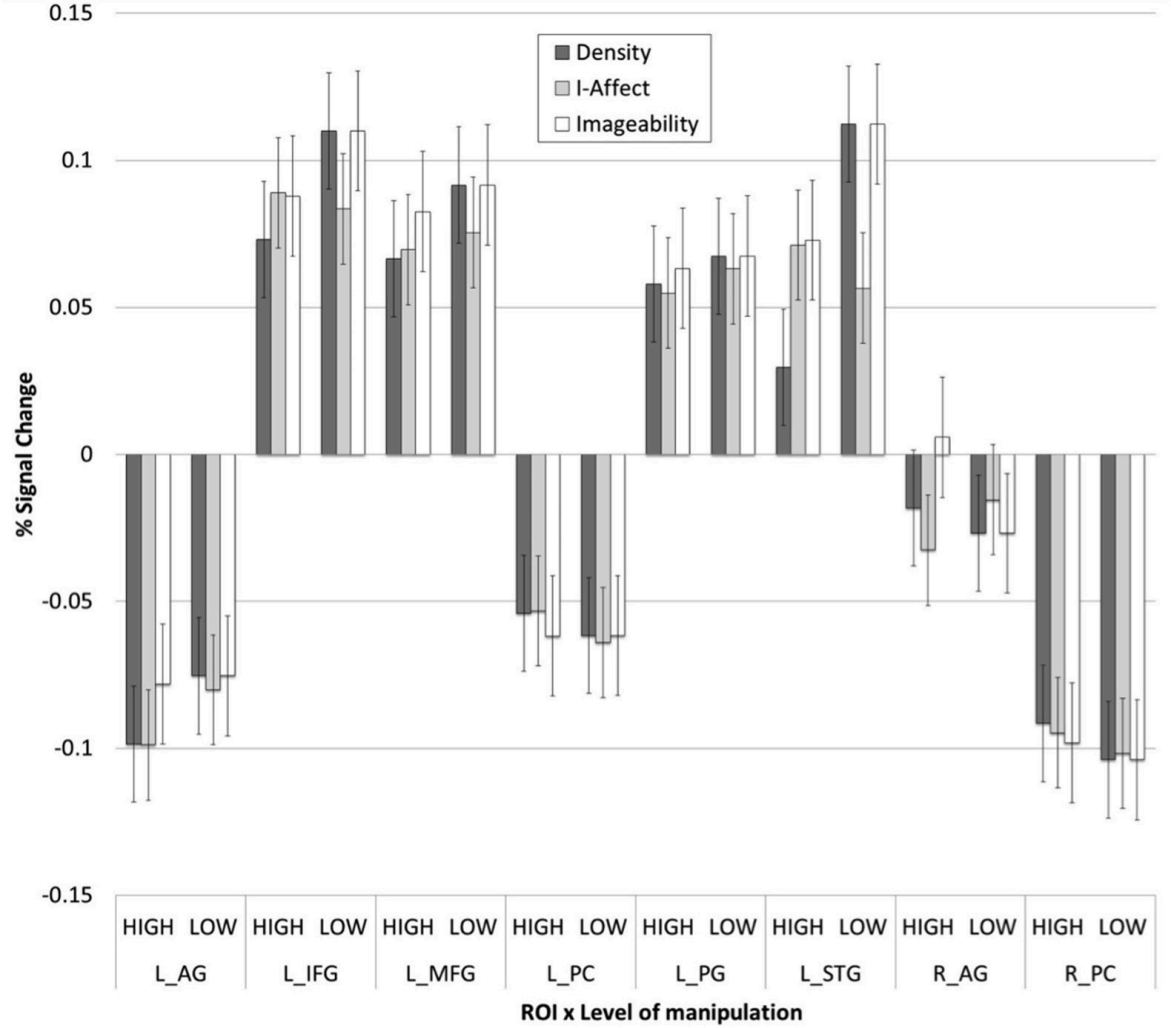
**The mean percent signal change (y-axis; ± SE) extracted from each region of interest and condition (x-axis)**.

The results are straightforward: for each ROI, the mean percent signal change was not statistically different across the conditions (imageability, DENSITY, and I-AFFECT; see Figure 7).

### Graphical model analysis

**Figure 8:**
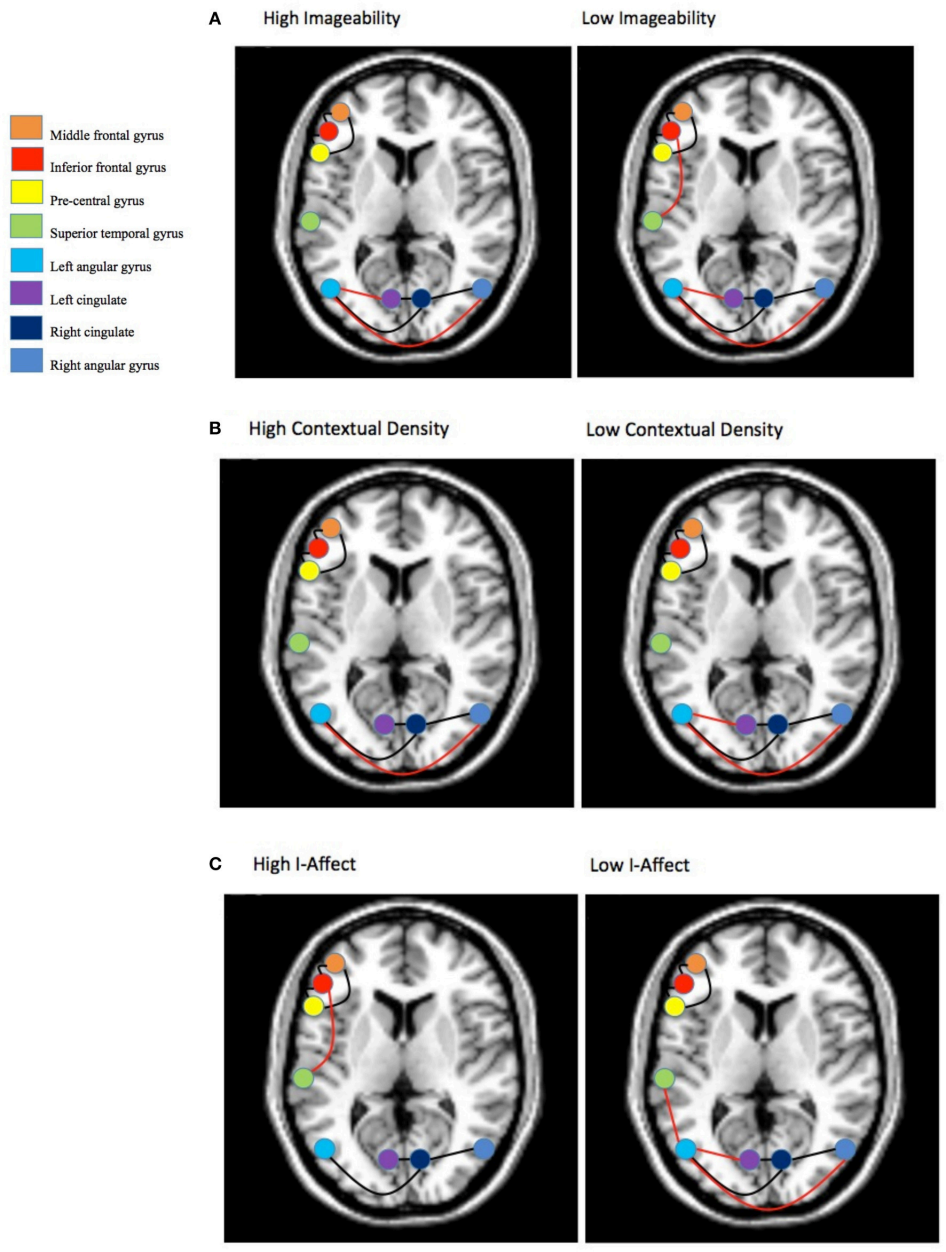
**Group graphical model for: (A) Imageability, (B) Context density, and (C) Affective valence**. Connections represent partial correlations that have bootstrap replication probability *p* ≥ 0.500 at the individual level and *p* < 0.05 for the group. Slice is at *z* = 50. Connections in black are part of the commonly shared network. Connections shown in red are unique to a particular construct.

The results of the graphical model analysis are presented in Figure 8. Across all conditions (high/low x imageability/I-AFFECT/DENSITY) a common network was found (shown with black edges in Figure 8) that was comprised of two sub-networks with six connections: an anterior network including the middle frontal gyrus, inferior frontal gyrus and pre-central gyrus and a posterior network that included connections among bilateral angular gyrus and bilateral cingulate.

Beyond the common network, imageability (Figure 8A) showed additional connections (shown in red in Figure 8) between the left and right angular gyri and between the left angular gyrus and the left cingulate. One additional connection for the low imageability condition was found and included the inferior frontal gyrus—superior temporal gyrus connection.

DENSITY (Figure 8B) showed similar connections to imageability with a somewhat reduced network. There were no additional and/or unique connections over and above those reported for imageability.

I-AFFECT (Figure 8C) showed a similar and reduced network to that reported for imageability. However, the superior temporal gyrus was connected in both the high I-AFFECT condition (to the inferior frontal gyrus) and the low I-AFFECT condition (to the left angular gyrus). Low imageability words are generally associated with high affect. The difference between the low imageability and high I-AFFECT networks is two additional connections in the low imageability network, between the left and right angular gyri and the left cingulate. The difference between the high imageability and low I-AFFECT networks is a single extra connection in the low I-AFFECT network, between the left angular gyrus and the left superior temporal gyrus.

### Discussion

Overall, we found the mean activity associated with high/low imageability, DENSITY, and I-AFFECT showed similar patterns of activation. Consistent with Binder et al. (2005), we found left hemisphere dominant activity for all conditions in the inferior frontal gyri (specifically the insula) and pre and post-central gyri. We found activity specifically associated with high imageable words in the left angular gyrus, which is consistent with activity reported by Binder et al. (2005), who reported activity at −37, −74, 26, compared to −50, −52, 6 in our data. They also reported activity in the right angular gyrus, left superior temporal pole and in posterior medial regions in both hemisphere. While we did not find these same clusters of activation, which is arguably a result of the carefully matched stimuli in the current study, we did find significant connections between the right and left angular gyri and the posterior cingulate for all constructs through the graphical model analysis. These bilateral connections are consistent with the claims of Dual Process Theory. In general, the mean activation maps support our hypothesis that the construct of imageability can be encapsulated by DENSITY and I-AFFECT constructs.

This interpretation is further supported by both the ROI and graphical model analyses. The ROI analysis shows no signal changes attributable uniquely to imageability, since statistically indistinguishable changes are seen when the data are analyzed by either DENSITY or I-AFFECT. The network analysis provided evidence of two networks comprising five of the ROIs, whether the data are analyzed by imageability, DENSITY, or I-AFFECT. There were no connections that were unique to the imageability manipulation alone. These results emphasize the close similarity in gross activation patterns associated with the three dimensions when each one was controlled for differences on the other two.

## General discussion

Here we provide converging behavioral and neuroimaging evidence for the idea that there is a close relationship between the construct of imageability and the combined constructs of DENSITY and I-AFFECT. In the behavioral analysis, we replicated the main finding of Westbury et al. (2013), that imageability effects are attenuated or eliminated when stimuli are controlled DENSITY and I-AFFECT. Although entering DENSITY and I-AFFECT in to our models did not eliminate a reliable effect for imageability (see Table 2), the effects on LDRT that are attributable to word imageability judgments are very small (an estimated 9 ms imageability effect) when DENSITY and I-AFFECT are controlled for. We also showed this finding was replicable with an independent data set, using RTs from the ELP (Balota et al., 2007).

In the fMRI analyses, we showed (1) similar (but not identical; see Figure 6 and Table 4) mean activity for all three constructs; (2) that a direct comparison of percent signal change activity in eight ROIs showed no differences among the three constructs (Figure 7); and (3) that the networks underlying the connectivity among those eight ROIs were very similar (though not identical; see Figure 8) for all three constructs.

Together, these findings support our hypothesis that human imageability judgments are systematically selected along the dimensions of DENSITY and I-AFFECT, resulting in a confound that makes it impossible to know whether effects that have been routinely attributed to imageability are actually due to imageability. We focus our discussion on the impact these findings have for theoretical advancements of models of imageability.

One question that arises whenever faced with correlated constructs is: which construct(s) should have ontological priority? This is especially relevant in this case, where we propose to replace a single construct with high face validity (i.e., imageability) with two abstract constructs from a computational co-occurrence model of semantics. In their classic paper on construct validity, a founding document of modern psychometrics, Cronbach and Meehl (1955) argued that constructs are validated only as “principles of inference,” i.e., in terms of their utility for licensing empirically-grounded inferences from empirically-accessible signs. For example, Baayen (2010) argued against the existence of word frequency effect, the best known predictor of almost every measure of lexical access, on the basis that “word frequency effect is an epiphenomenon of learning to link form to lexical meaning” (p. 2). Correlated properties of word co-occurrence that are directly stored by a Naïve Discriminant Learning model of language processing (Baayen et al., 2011) can account for all the variance that has normally been attributed to lexical frequency, which is not directly stored by the model. The correlated properties therefore function better as principles of inference, since a well-defined, theoretically-motivated model allows them, but not word frequency, to be directly measured.

Imageability judgments can be used as a principle of inference, since selecting words according to those judgments has many well-documented predictable behavioral consequences. However, the construct of imageability is not a good principle of inference, for three reasons. One is that it is not directly measureable but has to be estimated by humans. As a result, experiments that manipulate imageability are really only matching human intuitions about imageability to human performance, which does not move us forward in terms of explaining human behavior. Such experiments have made the mistake of treating human judgments as an independent variable when they are in fact *a dependent variable*, the very thing we need to explain.

The second reason that the imageability construct is problematic is that it obscures some distributional asymmetries in correlated predictors that are demonstrably relevant to the behavioral effects attributed to imageability, as we have shown this paper and in Westbury et al. (2013). Since DENSITY and I-AFFECT are directly calculable from a well-defined model supported by much other data, and since they allow more fine-grained predictions of behavior than imageability does (see Figures 3, 4), they are better principles of inference than imageability. Following the classical psychometric logic of Cronbach and Meehl (1955), they are therefore, also better constructs.

The final reason that imageability does not allow clear inferences is that it appears to have almost no consistent underlying neural substrate. As discussed in the introduction, previous imaging studies of imageability have yielded inconsistent results. We have added to the confusion by reporting results that are at best partially consistent with earlier studies using exactly the same task. Manipulating imageability does not seem to cleave the brain neatly into clear and distinct systems. Such variability suggests that we have not yet defined imageability as a biologically-relevant variable. Our approach has the advantage of being grounded in two theoretically-motivated constructs whose values were obtained from a well-defined computational model of semantics, thereby sidestepping the problematic practice of trying to explain the mind merely by correlating differences in human opinions with differences in human behavior.

There may of course be other constructs correlated with I-AFFECT, DENSITY, and imageability that will prove to be more useful for explaining imageability effects, for any one of a number of reasons: because they are simpler, more biologically plausible, easier to compute, neurologically clearer, more aesthetically pleasing, better integrated with a wider range of theory, and so on. We have explained above why I-AFFECT and DENSITY are useful constructs, how they can illuminate some problems with relying on imageability judgments as an explanatory variable, and how they are largely consistent in their neurological effects with each other and with imageability judgments themselves. However, nothing we have shown can prove that we have definitively identified “the true source” of imageability effects.

## Conclusion

Our behavioral and neuroimaging findings may seem to clearly support Context Availability Theory in showing that abstract words are more context-bound. However, it has been argued (Prinz, 2002; Dellantonio et al., 2014) that context effects are not incompatible with Paivio's Dual Code Theory, since Paivio's theory predicts that concrete words will be relatively impervious to manipulations of lexical variables such as co-occurrence density, “because performance is already near ceiling levels when concrete words are presented in isolation” (Prinz, 2002, p. 132). Against this interpretation, we note that we have not claimed that contextual density has a larger behavioral effect in abstract words than in concrete words, but rather that the two types of words have distinct values along this dimension (see Figure 1). This claim seems more compatible with Context Availability Theory than with the claim that lexical effects are simply not apparent for concrete words because those words are accessed quickly due to their associations with sensory experience. On the other hand, our finding of a network of bilateral regions underlying imageability effects is compatible with Dual Process Theory.

Although our data do not speak directly to the issue, we speculate that conceptualizing imageability effects in terms of lexical variables may help explain why patients who show no evidence of impaired perception of visuoperceptual semantic features (a finding that is problematic for Dual Process Theory) may nevertheless show abstract word *sparing* following brain damage (see discussion in Papagno et al., 2009).

Given that DENSITY and I-AFFECT are manipulable variables, they should form the constructs of interest in explaining imageability effects. In contrast, imageability is a subjective rating that should be measured and explained. Such a shift in the experimental perspective will aid in the further advancement of models of imageability.

## Author contributions

CW designed the experiments and stimuli, conducted the behavioral experiments, and took the lead role in writing the manuscript. JC conducted and wrote up the fMRI experiments, as well as contributing further to the manuscript. IC conducted the graphical model analysis, as well as contributing further to the manuscript.

## Funding

This work was funded by a Discovery Grant from the Natural Sciences and Engineering Research Council of Canada (NSERC; grant number: 250018-2013) to the lead author. Funding for IC was provided by the Pearson Faculty Fellowship, Alberta School of Business and Alberta Health Services (AHS) Grant. This research was also partially supported by the Natural Sciences and Engineering Research Council (NSERC; grant number: 386617-2012) of Canada in the form of a research grant to JC.

### Conflict of interest statement

The authors declare that the research was conducted in the absence of any commercial or financial relationships that could be construed as a potential conflict of interest.

## Footnotes

^1^Over 23,162 words, the measure correlates with computationally-derived estimates of valence from Westbury et al. (2015) at *r* = −0.33 (*p* < 0.00001) and with estimates of arousal from the same paper at *r* = 0.60 (*p* < 0.00001).

^2^Rex toolbox Retrieved from http://gablab.mit.edu/index.php/research/95-gablab-site/gablab/people/swg
