# Supplementary material for: Imaging Imageability: Behavioral Effects and Neural Correlates of Its Interaction with Affect and Context
Source: Front Hum Neurosci. 2016 Jul 15;10:346. doi: 10.3389/fnhum.2016.00346 (PMC4945641; doi:10.3389/fnhum.2016.00346)
Supplement: Supplementary file 1 [file DataSheet1.docx]

**Appendix 1: Sources of the basic affect terms used a predictors**

Wundt’s (1896) proposed that affect consisted of three basic axes: one for evaluation (‘*pleasant/unpleasant’*), one for arousal (*‘excitement/depression’*) and one for attention (*‘tension/relaxation’*).

Osgood, Suci, & Tannenbaum (1957) used factor analysis on a large set of affective ratings.to derive three binary dimensions: ‘*good/bad*’, ‘*active/passive*’, and ‘*strong/weak’*.

Tomkins (1962, 1963) proposed eight basic emotions (ignoring his non-lexicalized ninth emotion, *dismell* or reaction to a bad smell), defining the dimensions with two terms each, the first for the milder version of the affect and the second for a stronger related emotion: *‘interest/excitement’, ‘enjoyment/joy’, ‘surprise/startle’, ‘distress/anguish’, ‘fear/terror’, ‘shame/humiliation’, ‘contempt/disgust’*, and *‘anger/rage’*.

Ekman, Sorenson, and Friesen (1969) proposed seven basic emotions: *happiness, surprise, fear, sadness, anger,* and *disgust*. Ekman (1999) extended this list with an additional eleven terms: *amusement, contempt, contentment, embarrassment, excitement, guilt, pride, relief, satisfaction, pleasure*, and *shame*.

Plutchik (1980) derived eight primary emotions from consideration of evolutionarily adaptive emotions relevant across species: *anger, fear, sadness, disgust, surprise, anticipation*, and *joy*.

Building on the four basic emotions (*expectancy, rage, fear*, and *panic*) he had originally argued for in 1982, Panksepp (2005) proposed that neural hard-wiring in the mammalian brain underlay seven primary emotions: *seeking, fear, rage, lust, care, panic*, and *play*.

Robinson, Storbeck, Meier, & Kirkeby’s (2004) analysis of affect focused on the general emotional characteristics of arousal and valence. They did not suggest a specific set of labels for basic emotions, so we took the general focus of their analysis and created a set of labels related to danger or the lack thereof, to arousal, and to approach/avoidance behavior: *approach, avoid, towards, away, to, from, evaluate, arouse, danger*, and *safe*.

Stevenson, Mikels, & James (2007) extended the affective norms (valence and arousal judgments) collected by Bradley & Lang (1999), for five emotions dimensions that they considered to be cross-culturally universal (following Ekman, 1993, and Levenson, 2003): *happiness, sadness, fear, disgust*, and *anger*.

Reizenzein (2009) proposed that most or all emotions could be expressed as variants of just a few: *happiness, unhappiness, hope, fear, surprise, disappointment*, and *relief*. Analysis of these emotions depends in Reizenstein’s theoretical framework on *desire* and *aversion*, so we also included these two terms.

Kassam, Markey, Cherkassy, Loewenstein, & Just (2013) looked at the neural correlates of nine basic emotions: *anger, disgust, envy, fear, happiness, lust, sadness*, and *shame*. They also listed 18 related terms, of which we added 17: *angry, enraged, disgusted, envious, jealous, afraid, frightened, happy, joyous, lustful, horny, proud, admirable, sad, gloomy, ashamed,* and *embarrassed*. Their term *revulsed* did not appear in the HiDEx dictionary and was not included in our list.

**Appendix 2: All word stimuli**

| STIM | LEN | ON | LNFREQ | zIMG | zI-AFFECT | zDENSITY | IMG-CAT | I-AFFECT-CAT | DENSITY-CAT |
| --- | --- | --- | --- | --- | --- | --- | --- | --- | --- |
| alley | 5 | 5 | 1.12 | 1.12 | 1.25 | 1.08 | High | High | High |
| amend | 5 | 3 | 0.81 | -0.61 | -0.79 | -0.99 | Low | Low | Low |
| anchor | 6 | 0 | 1.50 | 1.03 | 0.91 | -0.79 | High | High | Low |
| apathy | 6 | 0 | 0.91 | -0.54 | -2.64 | 0.77 | Low | Low | High |
| arise | 5 | 3 | 2.21 | -0.89 | -2.19 | -0.84 | Low | Low | Low |
| array | 5 | 1 | 2.99 | -0.75 | 0.53 | -0.79 | Low | High | Low |
| astute | 6 | 0 | 0.72 | -1.52 | -2.48 | -0.78 | Low | Low | Low |
| basin | 5 | 3 | 0.97 | 0.68 | 1.30 | 1.13 | High | High | High |
| bean | 4 | 15 | 1.42 | 1.17 | 1.23 | 0.96 | High | High | High |
| bench | 5 | 6 | 1.98 | 1.39 | -0.81 | -0.83 | High | Low | Low |
| bland | 5 | 8 | 0.57 | -1.13 | -0.88 | 1.11 | Low | Low | High |
| bleed | 5 | 4 | 1.23 | 1.11 | -0.83 | -0.74 | High | Low | Low |
| bliss | 5 | 2 | 1.19 | -1.11 | 0.94 | 1.16 | Low | High | High |
| bully | 5 | 12 | 2.06 | 0.84 | -1.08 | 0.52 | High | Low | High |
| burden | 6 | 0 | 2.45 | 0.50 | -1.20 | 0.69 | High | Low | High |
| burst | 5 | 6 | 1.76 | -0.77 | 0.84 | 1.12 | Low | High | High |
| cache | 5 | 1 | 2.15 | -1.34 | 0.68 | -0.74 | Low | High | Low |
| cease | 5 | 6 | 2.32 | -1.45 | -0.90 | -0.78 | Low | Low | Low |
| chap | 4 | 10 | 1.25 | 0.64 | -0.71 | -0.80 | High | Low | Low |
| clad | 4 | 8 | 0.09 | -1.96 | 1.81 | -0.88 | Low | High | Low |
| cloak | 5 | 3 | 0.72 | 0.71 | -0.60 | 0.93 | High | Low | High |
| coral | 5 | 4 | 1.16 | 1.03 | 1.63 | -0.74 | High | High | Low |
| cougar | 6 | 0 | -0.95 | -0.76 | 0.60 | 0.96 | Low | High | High |
| coyote | 6 | 0 | 0.83 | -0.59 | 0.80 | 1.01 | Low | High | High |
| cream | 5 | 3 | 2.55 | 1.20 | 1.90 | -0.87 | High | High | Low |
| crook | 5 | 4 | 1.26 | 0.62 | -0.97 | 1.15 | High | Low | High |
| crutch | 6 | 3 | 0.30 | 1.19 | -0.72 | -1.04 | High | Low | Low |
| curb | 4 | 5 | 1.31 | 1.04 | -1.27 | 0.54 | High | Low | High |
| curt | 4 | 11 | 1.85 | -1.70 | -0.83 | 1.17 | Low | Low | High |
| czar | 4 | 3 | 0.07 | -1.02 | -0.53 | 0.58 | Low | Low | High |
| deaf | 4 | 5 | 1.62 | -0.94 | -0.75 | -0.78 | Low | Low | Low |
| differ | 6 | 2 | 2.23 | -0.83 | -2.32 | -0.79 | Low | Low | Low |
| dorm | 4 | 9 | 2.57 | 1.25 | 1.25 | -0.80 | High | High | Low |
| dread | 5 | 6 | 1.07 | -0.99 | -0.55 | 1.14 | Low | Low | High |
| duct | 4 | 5 | 1.12 | -0.77 | 0.55 | 0.78 | Low | High | High |
| dusty | 5 | 8 | 1.00 | 0.56 | 2.26 | 1.16 | High | High | High |
| eager | 5 | 9 | 2.05 | -0.54 | -1.05 | 0.99 | Low | Low | High |
| earl | 4 | 4 | 2.12 | -0.53 | 1.13 | 0.72 | Low | High | High |
| epic | 4 | 2 | 1.53 | -1.11 | 0.94 | 1.15 | Low | High | High |
| expose | 6 | 0 | 2.59 | -0.97 | -1.32 | 0.80 | Low | Low | High |
| fabric | 6 | 0 | 1.85 | 0.81 | 1.19 | 1.14 | High | High | High |
| fancy | 5 | 3 | 2.68 | -0.60 | 0.92 | 1.06 | Low | High | High |
| flake | 5 | 5 | 0.40 | 0.68 | -1.01 | -0.79 | High | Low | Low |
| flaw | 4 | 12 | 1.75 | -1.11 | -1.73 | -0.84 | Low | Low | Low |
| flown | 5 | 4 | 1.39 | -0.51 | 1.33 | -0.75 | Low | High | Low |
| flung | 5 | 4 | -0.08 | -0.85 | 0.53 | -0.74 | Low | High | Low |
| forge | 5 | 4 | 1.39 | -0.94 | -1.92 | 1.09 | Low | Low | High |
| frown | 5 | 5 | -0.28 | 1.23 | -0.80 | -1.22 | High | Low | Low |
| giver | 5 | 8 | 0.23 | -0.53 | -1.04 | -0.74 | Low | Low | Low |
| gloss | 5 | 7 | 0.15 | 0.68 | -0.80 | -0.75 | High | Low | Low |
| grade | 5 | 8 | 2.87 | -0.74 | 1.17 | -0.96 | Low | High | Low |
| grass | 5 | 9 | 2.35 | 1.42 | 1.45 | 1.14 | High | High | High |
| grim | 4 | 9 | 1.11 | -1.19 | 0.57 | 1.15 | Low | High | High |
| hasty | 5 | 6 | 0.44 | -0.71 | -1.55 | 0.54 | Low | Low | High |
| hinge | 5 | 3 | 0.08 | 0.51 | -1.79 | 0.98 | High | Low | High |
| hoist | 5 | 6 | -0.17 | -1.02 | 0.61 | -0.75 | Low | High | Low |
| honey | 5 | 11 | 2.04 | 1.43 | 1.95 | 1.08 | High | High | High |
| hull | 4 | 15 | 1.20 | -0.77 | 1.00 | 1.15 | Low | High | High |
| hurdle | 6 | 3 | -0.11 | 1.26 | -0.90 | -0.91 | High | Low | Low |
| jeans | 5 | 6 | 1.39 | 1.87 | 0.92 | -0.75 | High | High | Low |
| knack | 5 | 3 | 0.06 | -1.45 | -2.47 | -1.08 | Low | Low | Low |
| knight | 6 | 0 | 2.43 | 1.51 | 0.67 | -0.78 | High | High | Low |
| layer | 5 | 14 | 2.60 | -0.56 | 0.98 | 1.07 | Low | High | High |
| leach | 5 | 8 | 0.47 | 0.51 | -0.67 | 1.16 | High | Low | High |
| lily | 4 | 5 | 1.35 | 0.86 | 0.77 | 1.14 | High | High | High |
| liquor | 6 | 0 | 1.08 | 1.14 | 1.44 | 1.10 | High | High | High |
| magnet | 6 | 0 | 1.19 | 0.88 | -0.98 | -0.83 | High | Low | Low |
| muzzle | 6 | 4 | 0.67 | 0.62 | -0.93 | -0.90 | High | Low | Low |
| nest | 4 | 14 | 1.48 | 1.23 | 1.50 | -0.83 | High | High | Low |
| oven | 4 | 5 | 1.74 | 1.46 | 1.02 | -0.84 | High | High | Low |
| plaza | 5 | 1 | 1.40 | -0.76 | 1.57 | 1.12 | Low | High | High |
| poop | 4 | 12 | 1.05 | 1.62 | 0.76 | -0.87 | High | High | Low |
| purse | 5 | 8 | 0.81 | 1.30 | 0.53 | -0.82 | High | High | Low |
| quart | 5 | 3 | 0.13 | 0.75 | 0.77 | -1.12 | High | High | Low |
| rabbi | 5 | 0 | 2.45 | 1.00 | -0.57 | -0.81 | High | Low | Low |
| rabbit | 6 | 2 | 1.96 | 1.46 | 1.78 | 1.14 | High | High | High |
| remark | 6 | 0 | 1.95 | -1.01 | -1.33 | -0.74 | Low | Low | Low |
| roast | 5 | 5 | 1.02 | 1.10 | 1.12 | -0.84 | High | High | Low |
| screw | 5 | 3 | 2.63 | 1.36 | -0.50 | 1.12 | High | Low | High |
| sect | 4 | 6 | 1.39 | -1.62 | -0.59 | 1.14 | Low | Low | High |
| seldom | 6 | 0 | 1.81 | -1.81 | -1.37 | 0.73 | Low | Low | High |
| sequel | 6 | 0 | 0.99 | -1.48 | 0.53 | -0.88 | Low | High | Low |
| sham | 4 | 15 | 1.22 | -1.19 | -1.14 | -0.75 | Low | Low | Low |
| slam | 4 | 16 | 1.65 | 0.66 | -0.52 | -0.98 | High | Low | Low |
| slang | 5 | 5 | 1.06 | -0.54 | 0.59 | -0.77 | Low | High | Low |
| slope | 5 | 4 | 1.32 | 0.88 | 1.10 | 0.96 | High | High | High |
| spark | 5 | 6 | 1.62 | 0.85 | -2.70 | 1.15 | High | Low | High |
| spawn | 5 | 2 | 0.62 | -0.77 | -1.17 | 1.08 | Low | Low | High |
| sphere | 6 | 0 | 1.83 | 1.07 | -0.86 | 1.15 | High | Low | High |
| spite | 5 | 9 | 2.22 | -0.65 | -1.95 | -0.74 | Low | Low | Low |
| steady | 6 | 2 | 2.08 | -0.65 | -0.86 | 1.10 | Low | Low | High |
| steer | 5 | 5 | 1.06 | 0.51 | -0.70 | 0.85 | High | Low | High |
| stench | 6 | 1 | 0.67 | -0.94 | 1.27 | -0.79 | Low | High | Low |
| stool | 5 | 3 | 0.38 | 1.28 | 0.66 | -0.75 | High | High | Low |
| strung | 6 | 3 | 0.11 | -1.36 | 0.70 | -0.86 | Low | High | Low |
| summit | 6 | 2 | 2.11 | 0.98 | -0.66 | 1.09 | High | Low | High |
| sunk | 4 | 14 | 0.99 | -0.77 | 1.03 | -0.84 | Low | High | Low |
| surge | 5 | 4 | 1.41 | -0.94 | -1.05 | -0.74 | Low | Low | Low |
| swamp | 5 | 3 | 2.71 | 1.33 | 1.49 | 1.16 | High | High | High |
| swing | 5 | 9 | 2.58 | 1.19 | -0.81 | 1.16 | High | Low | High |
| tailor | 6 | 5 | 2.96 | 0.50 | -0.92 | -0.80 | High | Low | Low |
| theme | 5 | 3 | 2.57 | -0.64 | 0.57 | 1.05 | Low | High | High |
| throne | 6 | 2 | 1.72 | 1.45 | 0.83 | -0.91 | High | High | Low |
| tiger | 5 | 6 | 1.95 | 1.41 | 0.77 | 1.17 | High | High | High |
| tomato | 6 | 0 | 1.00 | 1.45 | 1.45 | -0.80 | High | High | Low |
| topped | 6 | 12 | 0.63 | -0.94 | 1.40 | -0.78 | Low | High | Low |
| tract | 5 | 5 | 0.93 | -1.45 | 0.70 | 1.09 | Low | High | High |
| trait | 5 | 5 | 1.22 | -1.28 | -2.10 | -0.78 | Low | Low | Low |
| tram | 4 | 9 | 0.44 | -1.28 | 0.75 | 1.00 | Low | High | High |
| trek | 4 | 4 | 2.23 | -1.28 | 1.31 | -1.00 | Low | High | Low |
| trend | 5 | 2 | 2.63 | -0.77 | -1.42 | 0.78 | Low | Low | High |
| twelve | 6 | 0 | 2.43 | 0.89 | 0.60 | 1.15 | High | High | High |
| umpire | 6 | 1 | 0.07 | 1.12 | -1.01 | -0.81 | High | Low | Low |
| vault | 5 | 3 | 0.98 | 0.62 | 0.53 | 1.09 | High | High | High |
| vent | 4 | 14 | 1.26 | 0.85 | -1.10 | 0.99 | High | Low | High |
| virtue | 6 | 0 | 2.28 | -1.03 | -1.92 | -0.76 | Low | Low | Low |
| vista | 5 | 1 | 2.03 | -1.20 | 0.52 | -0.85 | Low | High | Low |
| warmth | 6 | 0 | 0.89 | 0.83 | -1.04 | 1.05 | High | Low | High |
| warp | 4 | 12 | 1.03 | -0.85 | -0.55 | 1.14 | Low | Low | High |
| whip | 4 | 9 | 1.90 | 0.93 | -0.77 | -0.83 | High | Low | Low |
